# A streamlined proximity extension assay using POEGMA polymer-coated magnetic beads for enhanced protein detection

**DOI:** 10.3389/fbioe.2024.1462203

**Published:** 2024-11-21

**Authors:** Jiumei Hu, Pengfei Zhang, Fangchi Shao, Tza-Huei Wang

**Affiliations:** ^1^ Department of Mechanical Engineering, Johns Hopkins University, Baltimore, MD, United States; ^2^ Department of Biomedical Engineering, Johns Hopkins University, Baltimore, MD, United States; ^3^ Institute for NanoBioTechnology, Johns Hopkins University, Baltimore, MD, United States

**Keywords:** proximity extension assay, POEGMA polymer, magnetic beads, highly sensitive, streamlined

## Abstract

The detection of protein biomarkers presenting at low concentrations in biological fluids is essential for disease diagnosis and therapeutic monitoring. While magnetic beads-based solid-phase immunoassays have shown promise in achieving high sensitivity for detecting low-abundance proteins, existing protocols suffer from limitations such as the cumbersome need for bead blocking and washing steps to minimize adsorption of non-specific biomolecules. These extra requirements lead to increased assay complexity and the risk of procedural errors. In this study, we present a streamlined magnetic proximity extension assay (MagPEA) using poly (oligo (ethylene glycol) methacrylate) (POEGMA)-coated beads. The polymer brush on bead surface, on the one hand, provides an effective mechanism for repelling non-specifically bound biomolecules that contribute to background signal generation without performing any bead blocking and washing steps. On the other hand, it facilitates the immobilization of capture antibodies on bead surface by simply embedding the antibodies onto the porous polymer under vacuum. Using the human inflammatory factor IL-8 as a demonstration, we show that the incorporation of POEGMA beads into MagPEA workflow significantly simplifies assay procedure while maintains high sensitivity.

## Introduction

Proteins in biological fluids, such as serum, constitute a vast and largely unexplored reservoir that contains important biological information. Effective detection of these protein biomarkers therefore holds tremendous utility across various scenarios, such as disease diagnostics and therapeutic monitoring (Landegren et al., 2018; Landegren and Hammond, 2021). However, the full translation of such utility into clinical practice is hindered by the challenge that some protein biomarkers are present at exceedingly low concentrations (Cohen and Walt, 2019; Mao et al., 2021; Duffy, 2023). Conventional methods, such as enzyme-linked immunosorbent assay (ELISA) and Western blot, while useful in many aspects, often suffer from limited sensitivity in detecting these low-abundance biomarkers (Tsai et al., 2016; Tsai et al., 2018).

As such, the pursuit of heightened sensitivity in protein biomarker detection has fueled the development of numerous innovative methods and cutting-edge technologies. One prominent strategy involves the utilization of antibody-immobilized magnetic beads in solid-phase immunoassays, which leverage the inherent advantages of magnetic beads to achieve sensitive and precise protein measurements (Darmanis et al., 2010; Rissin et al., 2010; Nong et al., 2013; Yelleswarapu et al., 2019; Lyu et al., 2021; Yi et al., 2022; Zhang et al., 2022b). First, magnetic beads have a high surface-to-volume ratio and can be easily resuspended in a solution with mixing, allowing for higher binding capacity and more efficient capture of specific protein analytes (Chang et al., 2012). Second, magnetic beads offer an effective mechanism for extensive washing during the immunoassay procedure. Using external magnetic field, these beads can be easily separated from complex sample matrices, facilitating the efficient removal of interfering substances that result in background signal generation. Moreover, magnetic beads contribute to improved sensitivity in immunoassays via efficient target enrichment.

A notable example of highly sensitive magnetic beads-based immunoassay is the Single Molecule Array technology (SiMoA), also known as digital ELISA (dELISA) (Rissin et al., 2010; Rissin et al., 2011). dELISA utilizes magnetic beads to capture proteins, labels proteins via the formation of immunocomplexes, and then digitizes individual beads carrying the immunocomplexes into femtoliter-sized microwells for localized signal amplification with single molecule resolution. In the past decade, dELISA has made significant technological advancements (Cohen et al., 2020; Wu et al., 2020; Wu et al., 2022), making it the current gold standard for ultra-sensitive protein detection. Other methods, such as magnetic beads-based proximity ligation assay (PLA) (Darmanis et al., 2010; Nong et al., 2013), also demonstrated higher sensitivity compared to its homogenous assay counterpart. A more recent advancement is the magnetic beads-based proximity extension assay (MagPEA) (Zhang et al., 2022b), which exhibits superior sensitivity close to that of dELISA. Both the solid-phase PLA and the MagPEA utilize the magnetic beads to capture target proteins, and then employ a pair of antibody-oligo conjugates, commonly referred to as PLA or PEA probes, to further recognize the captured proteins. When the two PLA or PEA probes bind onto the same protein, they are brought into close proximity, facilitating efficient ligation or extension of oligo tails to generate the DNA templates for downstream PCR amplification.

Nevertheless, despite the advantages of magnetic beads-based solid-phase immunoassays in enabling sensitive protein detection, the protocols employed in these assays possess inherent limitations. One major drawback of these assays is the requirement for bead blocking and extensive washing steps to mitigate the adsorption of non-specific biomolecules onto bead surface (Fu et al., 2021). These additional procedures prolong the overall assay time and add complexity to the assay operation, raising the risk of procedural errors and limiting high-throughput analysis capabilities for processing a large number of samples simultaneously. Furthermore, the need for repeated washing steps can result in the loss of beads that contain immunocomplexes, leading to biased output of results (Kan et al., 2020).

In this work, we sought to build a streamlined and highly sensitive beads-based immunoassay that circumvents the need for bead blocking and extensive washing steps. To achieve this, we adapt the MagPEA approach to incorporate magnetic beads coated with poly (oligo (ethylene glycol) methacrylate) (POEGMA) polymer brushes, known for their strong antifouling properties that repel non-specifically bound biomolecules (Hucknall et al., 2009; Joh et al., 2017). These polymer brushes are synthesized using a Glucose Oxidase (GOx)-assisted, oxygen-tolerant Activators Regenerated by Electron Transfer Atom Transfer Radical Polymerization (ARGET-ATRP) technique under ambient environment (Navarro et al., 2019). Our testing on the human inflammatory factor IL-8 using POEGMA bead-based MagPEA demonstrates that integrating POEGMA beads into the MagPEA workflow eliminates bead blocking and cumbersome washing steps and thus significantly speeds up the assay, while maintaining exceptional sensitivity comparable to our previously developed MagPEA. Furthermore, the incorporation of POEGMA brushes on the bead surface offers additional benefits as it acts as a porous substrate for immobilizing capture antibody via physical entanglement under vacuum without involving any covalent chemical reactions. Therefore, the combined benefits of simplified protocol and high sensitivity make the POEGMA bead-based MagPEA assay a promising candidate for highly sensitive and scalable protein detection across various applications in the future.

## Methods

### Materials

α-Bromoisobutyryl bromide (98%), Dichloromethane (DCM, anhydrous, ≥99.8%), Triethylamine (BioUltra, ≥99.5%), isopropyl alcohol (IPA, anhydrous, 99.5%), α-D-Glucose (anhydrous, 96%), GOx (from Aspergillus niger), Sodium pyruvate (BioXtra, ≥99%), Sodium bromide (NaBr, BioXtra, ≥99.0%), Copper(II) bromide (CuBr_2_, 99%), L-Ascorbic acid (BioXtra, ≥99.0%), Poly(ethylene glycol) methacrylate (POEGMA, average Mn 360, contains 500–800 ppm MEHQ as inhibitor), 1,1,4,7,10,10-Hexamethyltriethylenetetramine (HMTETA, 97%), tetrahydrofuran (THF, Anhydrous, 99.9%) and Aluminum oxide were purchased from Millipore Sigma. Amine-terminated Dynabeads (M-270, 2.8 μm in diameter, stock concentration: 2 × 10 ^ 9 beads/mL) was purchased from Thermofisher Scientific. A comprehensive list of all the reagents utilized in performing MagPEA can be found in our previously published paper (Zhang et al., 2022b).

### Initiator attachment

To incorporate the bromo-initiator, 100 μL of Dynabeads were first washed twice with PBS-Br buffer (PBS-Br buffer was prepared in the same way as PBS, except that the NaCl was replaced with NaBr) and dried in a vacuum chamber for 2 h at room temperature. The dried magnetic beads were resuspended in 1.25-mL anhydrous DCM and then transferred into a pre-dried glass vial. Next, 700 μL of Triethylamine and 370 μL of α-Bromoisobutyryl bromide were added sequentially into the vial. The vial was tightly sealed with a screw cap, covered with aluminum foil, and placed on an end-over-end rotator for 12 h at room temperature. Subsequently, the beads were extensively washed with ∼5 mL of DCM to remove the generated dark precipitates. The beads were then transferred into a 1.5 mL tube and washed twice with IPA followed by two washes with milli-Q water. Finally, the beads were resuspended in 200 μL of PBS-Br buffer and stored at 4°C before use.

### ARGET-ATRP on beads

To generate POEGMA polymer brushes on the beads, polymerization solutions were prepared as combinations of a 2 × glucose mixture and a 2 × monomer mixture. Specifically, a 500-μL 2 × glucose mixture is composed of 120 μL of 30% glucose, 110 μL of 10% sodium pyruvate, 10 μL of 5.0 kU/mL GOx in 260 μL of PBS-Br buffer. Meanwhile, a 500-μL 2 × monomer mixture was prepared by mixing 176 μL of OEGMA, 62 μL of 0.3 mg/mL L- Ascorbic acid, 11.8 μL of 10 mg/mL CuBr_2_, 0.2 μL of HMTETA ligand in 250 μL of PBS-Br buffer. Next, the 500-μL 2 × glucose mixture and 2 × monomer mixture was mixed with 50-μL initiator-attached magnetic beads in a 1.5 mL tube. The resulting solution was then subjected to ATRP reaction on an end-over-end rotator for different time periods at room temperature. After reaction, the tube was briefly centrifuged and placed onto a magnetic rack to pellet beads and remove supernatant. The beads were then washed 4 times with 200 μL of 50% THF in PBS-Br buffer to eliminate free polymers. Finally, the beads were resuspended in 50-μL PBS-Br buffer (beads concentration: 10^9^ beads/mL) and stored at 4°C before use.

### Capture antibody immobilization onto beads

The high-density POEGMA matrix on the surface of magnetic beads provide a substrate to physically immobilize capture antibodies onto the polymer brushes via vacuum desiccation without covalent coupling. In this process, 1 μg of antibody was used per 5 μL of POEGMA beads in a 20-μL PBS solution. The detailed calculation regarding the necessary quantity of antibody to achieve bead saturation can be found in the Supplementary Material. The beads suspension was then placed in a vacuum chamber at −100 kPa for 8 h, allowing the protein to be physically immobilized through the intertwining between antibody and the polymer chains. Subsequently, the beads were washed 3 times with 100 μL of washing buffer (0.05% Tween-20 in PBS) to remove unbound protein. Finally, antibody-coated beads were resuspended in PBS at a final concentration of 5 × 10^8^ beads/mL.

### Antibody-oligo conjugation

Conjugation between antibody and thiol-modified oligo was performed via Sulfo-SMCC mediated covalent coupling. Specifically, 14 μL of 1 mg/mL purified antibody solution was mixed with 2 μL of 3.33 mM Sulfo-SMCC. The mixture was then incubated at 4°C for 2 h with 3 intermittent mixing. Meanwhile, 2.6 μL of each oligos at a concentration of 500 μM (including oligo A and oligo B) was mixed with 4.4 μL of 40 mM DTT. The oligos were then reduced by incubating at 95°C for 2 min and 37°C for 1 h. Next, the activated antibody and reduced oligos were purified using 40 kDa and 7 kDa zeba desalting spin columns, and they were combined with a 10× molar excess of oligos to antibody. The resulting mixtures were further incubated at 4°C overnight to ensure complete conjugation. Subsequently, the antibody-oligo conjugates were purified with 40 kDa zeba desalting spin columns and concentrations were measured using Bicinchoninic acid (BCA) assay.

### MagPEA

In MagPEA, 2 immunobinding steps were involved and 5 × 10^5^ POEGMA beads were used for each reaction. During first immunobinding step, beads were resuspended in 100 μL of protein spiked in 20% Fetal Bovine Serum (FBS) and incubated at room temperature on an end-over-end rotator for 15 min to capture target protein. The beads were then pelleted on a magnetic rack to remove supernatant. During second immunobinding step, 20 μL of 5 nM PEA probe mixture in MagPEA buffer (1% BSA, 0.2 mg/mL salmon sperm DNA, 0.1% Tween-20, 150 mM NaCl, 0.05% dextran sulfate, and 5 mM EDTA in 1 × PBS) was added into the beads and incubated at room temperature on an end-over-end rotator for 15 min. Next, free PEA probes were eliminated by pelleting beads on the magnetic rack and the beads were resuspended in 10 μL of PCR buffer containing 1 × TaqMan gene expression master mix, 100 nM of forward and reverse primer, 600 nM of TaqMan probe, and 0.1 U/μL *Bst* 2.0 DNA polymerase. One-step extension-PCR was performed on Bio-Rad CFX96 Real-Time PCR System with a thermocycling program as follow: 95°C for 10 min, 15 s at 95°C and 60 s at 60°C for 50 cycles.

### Confocal microscopy

The fluorescence on POEGMA beads after incubation with FITC-BSA solution were measured using Zeiss LSM780 confocal microscope. Specifically, 2 μL beads at a concentration of 5 × 10^8^ beads/mL in PBS was added onto a 75 mm × 25 mm glass slide. An 18 mm × 18 mm glass coverslip was then put on top of the beads suspension to immobilize the beads. Beads were imaged using ×40 objective lens with a gain setting as 50. The fluorescence intensity of each bead was then analyzed using the adaptive thresholding in Fiji.

## Results

### Overview of POEGMA bead-based MagPEA

**FIGURE 1:**
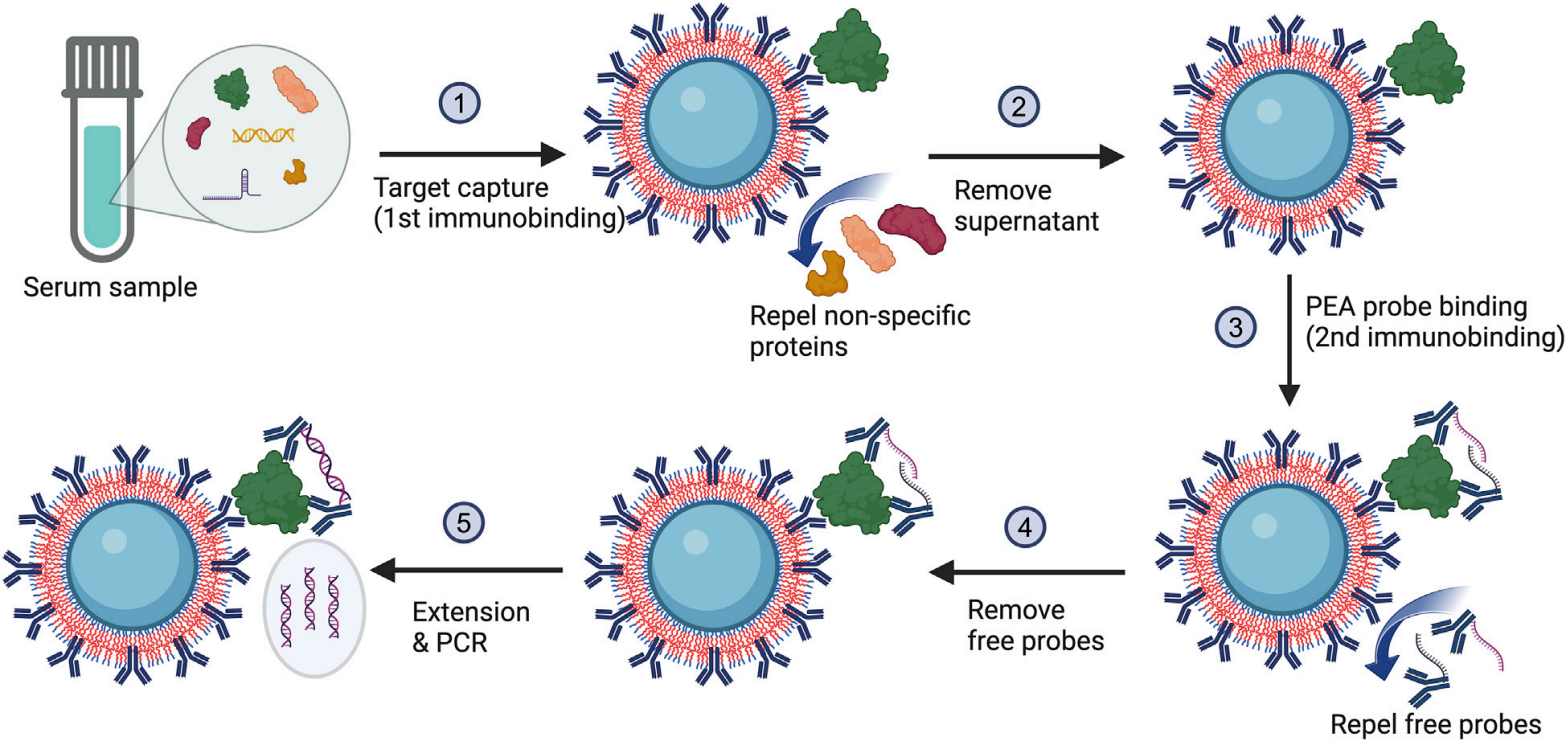
The MagPEA workflow using POEGMA beads eliminates bead blocking and repeated washing steps. During this process, the POEGMA beads immobilized with capture antibody first capture antigens in a sample. After pelleting beads to remove supernatant, a pair of PEA probes is added to further recognize the antigen. When the PEA probes simultaneously bind to the identical antigen, they are brought into close proximity with an elevated local concentration, facilitating the hybridization of the oligonucleotides to one another. Following hybridization, the oligonucleotides are enzymatically extended by DNA polymerase, generating double-stranded DNA template for PCR amplification.

Our POEGMA bead-based MagPEA involves capturing the target antigen to antibody-coated POEGMA beads (Figure 1, step 1), followed by adding a pair of antibody-oligo conjugates (PEA probes) to further recognize the target antigen (Figure 1, step 3). The two oligos on these PEA probes are designed with 5-bp complementary sequences on their free 3′ tails, allowing the tails to hybridize, extend, and then be amplified by downstream PCR (Figure 1, step 5) only when the two PEA probes are brought into close proximity after binding to the same antigen. Such a triple-binding requirement of the antibody to the target antigen ensures high assay stringency, thereby minimizing background signals for enhanced sensitivity.

In our previously developed MagPEA (Zhang et al., 2022b), we routinely conducted repeated washings immediately following the antigen capture and PEA probe binding (Figure 1, step 2 & 4) to remove any non-specific proteins and free PEA probes that might bind to the magnetic beads for reducing background signals. However, with the utilization of POEGMA beads, the polymer brushes on the bead surface act to repel the non-specific proteins and free PEA probes, effectively keeping them in the solution phase (Hucknall et al., 2009; Joh et al., 2017). As a result, the removal of these non-specific binding events can be simplified to merely aspirating the supernatant. Moreover, the pre-blocking of beads, another routine procedure in our previously developed MagPEA and other bead-based immunoassays, has also been eliminated with the use of POEGMA beads.

### Growing POEGMA polymer brushes on magnetic beads

**FIGURE 2:**
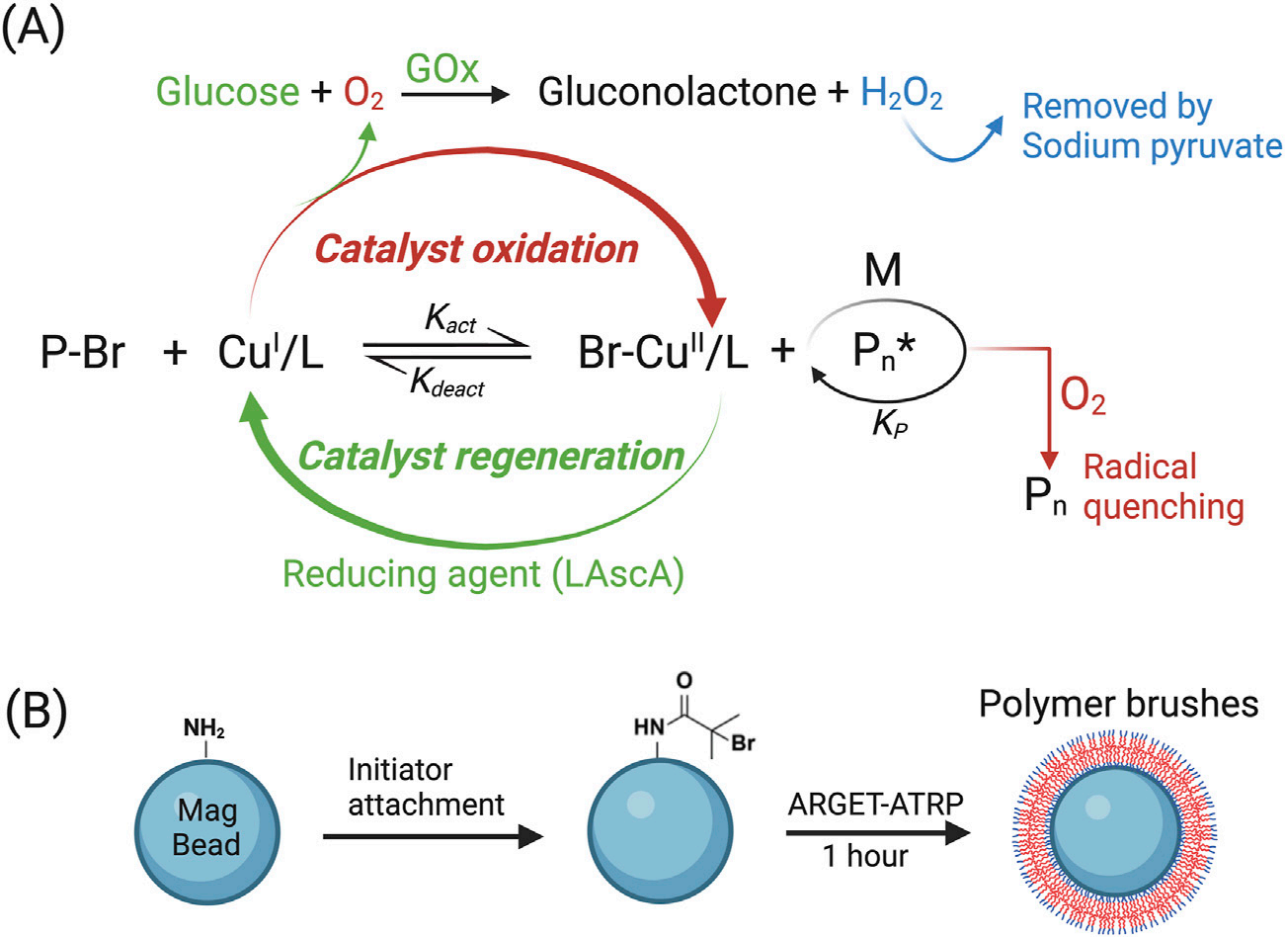
Utilizing ARGET-ATRP to generate POEGMA polymer on bead surface. **(A)** Diagram illustrating the principle of GOx-assisted ARGET-ATRP technique. **(B)** Initiator was attached onto amine-terminated magnetic beads before conducting the ARGET-ATRP reaction.

To synthesize POEGMA polymer brushes on magnetic beads, we employed the ARGET-ATRP technique, an oxygen-tolerant variant of traditional ATRP (Matyjaszewski, 2012; Matyjaszewski and Tsarevsky, 2014). ATRP is based on reversible redox reactions, involving a dynamic equilibrium between the dormant species (P-Br) and active radicals (Pn*) that is facilitated by a ligand-stabilized transition metal complex (typically copper (Cu), also known as catalyst) converting between its lower (Cu^I^/L) and higher oxidation states (Cu^II^/L). Traditional ATRP relies on a high concentration of Cu^I^/L to ensure a predominant rate constant of K_act_. This process involves the formation of an active radical by activating the dormant species, typically a carbon-centered radical with an attached halogen atom such as Br, in which the copper complex Cu^I^/L abstracts the halogen atom from the dormant species, resulting in the generation of an active radical that can initiate chain-growth polymerization when it reacts with monomers (M). However, even trace amounts of oxygen can hinder the polymerization process by rapidly oxidizing the metal complex Cu^I^/L into an inactive Cu^II^/L. Furthermore, oxygen molecules can interact with propagating radicals, leading to the termination of polymerization (Szczepaniak et al., 2020). Consequently, traditional ATRP must be conducted in anaerobic environment, limiting its broad use. In order to overcome the anaerobic constraint, ARGET-ATRP introduces an excess of reducing agent such as L-Ascorbic acid (LAscA) to convert all initially added Cu^II^/L and any Cu^II^/L accumulated due to catalyst oxidation back to Cu^I^/L, allowing the ATRP reaction to proceed in ambient environment. More recently, Glucose and GOx were reported to be incorporated into ARGET-ATRP to enhance oxygen scavenging, wherein GOx consumes oxygen while oxidizing glucose (Navarro et al., 2019). In our study, we opt for GOx-assisted ARGET-ATRP approach to generate POEGMA polymer brushes on magnetic beads (Figure 2A) (Matyjaszewski et al., 2006; Matyjaszewski et al., 2007). To initiate the process, we first introduced an initiator on bead surface by reacting the amino groups of beads with the acid bromide group of bromoisobutyryl bromide (Van Andel et al., 2017). This enables the growth of polymer brushes from the initiator through ARGET-ATRP reaction without the anaerobic environment restriction, thus significantly facilitating the anti-fouling coating (Figure 2B).

### Antifouling efficacy of POEGMA magnetic beads

**FIGURE 3:**
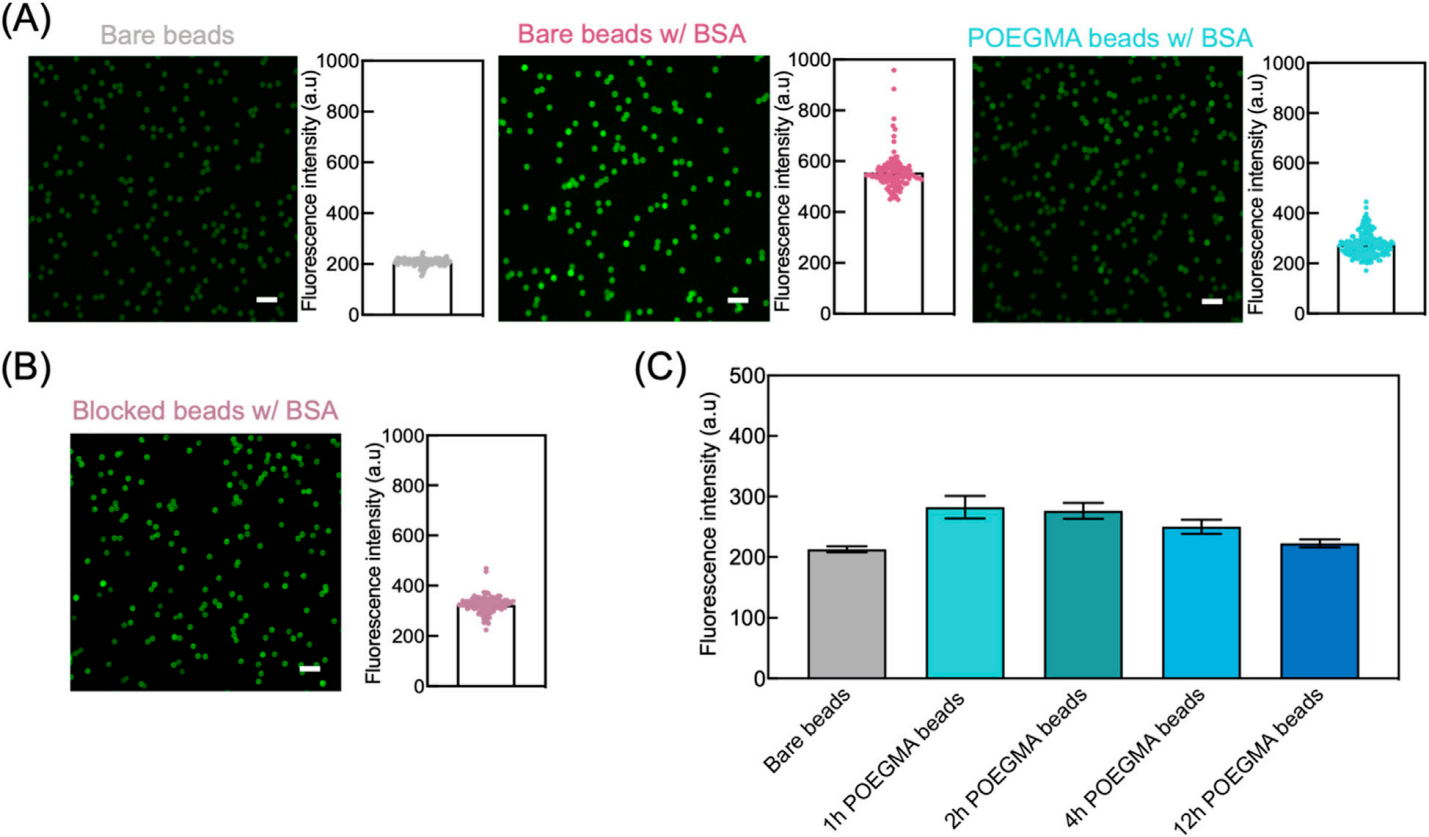
Enhanced antifouling performance of POEGMA beads. **(A)** We assessed the antifouling performance of POEGMA beads by incubating them with 1 mg/mL FITC-BSA solution on an end-over-end rotator at room temperature for 2 h. For comparison, we included bare beads as the negative control and bare beads incubated with FITC-BSA as the positive control. After incubation, the beads were washed three times using PBST and then imaged using ZEISS LSM780 confocal microscope. For each group, left images illustrate the fluorescence intensity of individual beads, and the fluorescence intensity of each bead was used to generate histograms on the right, where each dot represents an individual bead. Scale bar: 10 μm. **(B)** POEGMA beads showed better antifouling performance compared with the beads pre-blocked with 10% goat serum for 1 h, as indicated by the lower fluorescence intensity on beads after incubating with FITC-BSA. Scale bar: 10 μm. Each dot represents an individual bead. **(C)** POEGMA beads with increased ATRP reaction time showed decreased fluorescence intensities, suggesting improved antifouling capabilities. The error bars represent standard deviation from three replicate tests.

To evaluate the antifouling efficacy of POEGMA beads, we incubated beads with FITC-labelled BSA solution and then determined the degree of non-specific adsorption of BSA via on-bead fluorescence intensity measurement. Higher fluorescence intensity of beads indicates a higher degree of non-specific adsorption. Specifically, three groups of beads were tested in parallel including bare beads, bare beads incubated with FITC-BSA, and POEGMA beads incubated with FITC-BSA. The bare beads served as a negative control, while the bare beads incubated with FITC-BSA were used as a positive control to represent the typical non-specific adsorption level of biomolecules onto unmodified beads. The POEGMA beads were prepared by conducting ARGET-ATRP reaction for 1 h. After incubation, the beads were washed and then imaged under microscope. Our results showed a substantial decrease in fluorescence intensity for POEGMA beads compared to bare beads, indicating the effective suppression of non-specific adsorption of BSA using POEGMA beads (Figure 3A). We also compared the antifouling efficacy between POEGMA beads and blocked beads that are commonly used in bead-based immunoassays. Before incubating in FITC-BSA solution, the blocked beads were prepared by mixing bare beads with 10% goat serum. Our results showed that the POEGMA beads exhibited lower averaged fluorescence intensity compared to blocked beads (Student’s t-test, *p* = 0.019), suggesting a more effective suppression of non-specific protein adsorption by incorporating the POEGMA polymer brushes onto magnetic beads (Figure 3B).

Next, we assessed the antifouling efficacy of POEGMA beads subjected to various ARGET-ATRP reaction times, including 1, 2, 4, and 12 h. The beads were incubated with FITC-BSA solution, and antifouling performance was evaluated by measuring the on-bead fluorescence intensity. For comparison, we also measured the fluorescence intensity of bare beads to determine the background level. POEGMA beads exhibiting fluorescence intensities close to that of bare beads is indicative of superior antifouling properties. As shown in Figure 3C, all POEGMA beads demonstrated only a marginal increase in fluorescence intensity compared to bare beads, indicating effective suppression of non-specific FITC-BSA adsorption. Furthermore, a decreasing trend in fluorescence intensity with increasing ARGET-ATRP reaction times was observed, suggesting that POEGMA beads with thicker polymer layers exhibit enhanced antifouling capabilities (Figure 3C).

### Antibody immobilization onto POEGMA magnetic beads

Traditional bead-based immunoassays commonly employ covalent coupling chemistries to immobilize capture antibodies on bead surfaces. This process leverages the chemical modifications present on beads, such as amine and carboxyl groups, along with the reactive groups found on antibodies, to facilitate antigen capture. However, the hydroxyl termination group on POEGMA brushes presents a challenge for prevalent covalent coupling chemistries, making the immobilization of capture antibody onto beads difficult (Ma et al., 2006). To address this issue, we adopted a vacuum-assisted approach to physically embed antibodies onto bead surface by leveraging the porous structure of POEGMA brushes (Hucknall et al., 2009; Joh et al., 2017; Heggestad et al., 2021).

**FIGURE 4:**
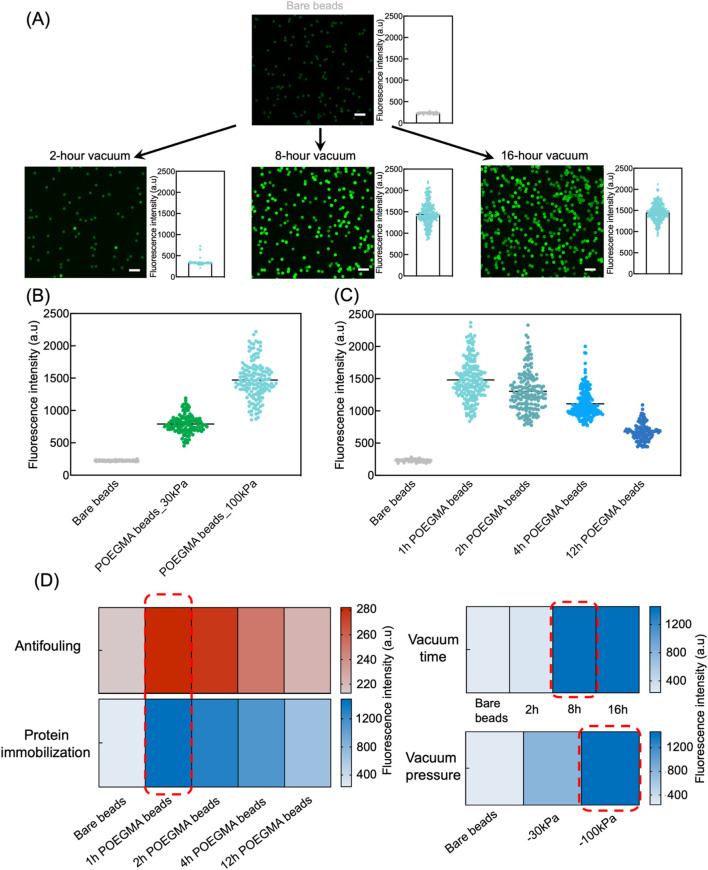
Efficient protein immobilization onto POEGMA beads via vacuum suction. **(A)** POEGMA beads with 1-hour ATRP reaction time were suspended in 20 μL of 0.125 mg/mL FITC-BSA solution. The beads suspensions were then subjected to 2-hour, 8-hour, and 16-hour desiccation in a vacuum chamber at a pressure of −100 kPa. Next, beads were washes three times using PBST. Fluorescence intensity of individual beads was measured under confocal microscope (left) and plotted as histograms (right), where each dot represents the fluorescence intensity of individual bead in the corresponding fluorescence images. Scale bar: 10 μm. **(B)** A high vacuum pressure at −100 kPa showed better protein immobilization efficiency in comparison to −30 kPa, as indicated by higher fluorescence intensity of POEGMA beads. Each dot represents the fluorescence intensity of an individual bead, and the distribution shows the intensity of all beads in a representative image. **(C)** POEGMA beads with increased ATRP reaction time showed decreasing protein immobilization efficiency. Each dot represents the fluorescence intensity of an individual bead, and the distribution shows the intensity of all beads in a representative image. **(D)** Heatmaps showing the final conditions selected (in red rectangle), including ATRP time for polymer growth, vacuum time and pressure for protein coating, to prepare the POEGMA beads.

To assess the effectiveness of vacuum-assisted approach for antibody immobilization, FITC-BSA was used as a model protein. The on-bead fluorescence intensity was measured to determine the immobilization efficiency, with higher fluorescence intensity indicating a higher amount of immobilized protein. We first investigated the required vacuum duration for efficient protein immobilization. Specifically, POEGMA beads generated via ARGET-ATRP reaction for 1 h were mixed with FITC-BSA solution, and then exposed to vacuum at a pressure of −100 kPa for 2 h, 8 h, and 16 h. Among the tested conditions, POEGMA beads subjected to a 2-hour vacuum suction showed low protein immobilization efficiency, as evidenced by the slight increase of fluorescence intensity compared to bare beads. In comparison, POEGMA beads exposed to vacuum for 8 h exhibited strong fluorescence intensity, indicating successful protein immobilization. Further extending the vacuum time to 16 h did not improve the protein immobilization density on beads (Figure 4A). Therefore, we opted for an 8-hour vacuum duration to prepare the POEGMA beads immobilized with capture antibodies for further experiments. Our results also indicated that a higher vacuum pressure at −100 kPa resulted in significantly greater protein immobilization efficiency compared to a lower vacuum pressure at −30 kPa (Figure 4B).

Next, we investigated protein immobilization efficiency on POEGMA beads that underwent varying ARGET-ATRP reaction times, including 1, 2, 4, and 12 h. Interestingly, we observed significant decrease in fluorescence intensity on beads with longer ARGET-ATRP reaction times, indicating a compromised protein immobilization efficiency due to thicker polymer brushes (Figure 4C; Supplementary Figure S1). This observation could possibly be attributed to the decreased porosity and increased repellence of POEGMA beads to proteins as the brushes thicken. Considering the intricate interplay between antifouling efficacy and protein immobilization efficiency of POEGMA beads, we finally determined the optimal ARGET-ATRP reaction time to be 1 h.

### MagPEA optimization and characterization using POEGMA magnetic beads

**FIGURE 5:**
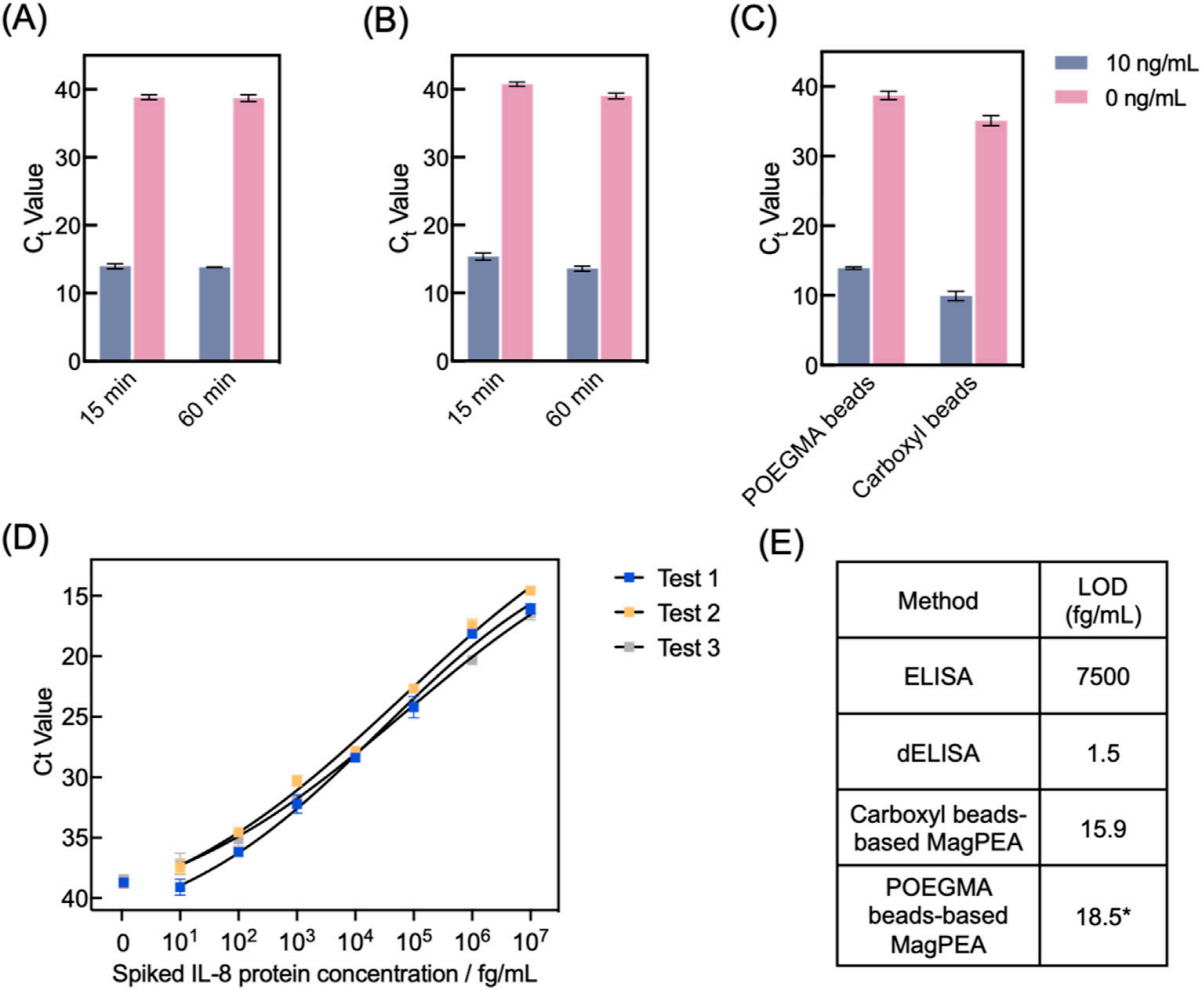
POEGMA beads-based MagPEA optimizations and LOD characterization target IL-8 protein. **(A)** We tested 15-minute and 1-hour immunobinding for protein capturing by POEGMA beads, and they showed similar signal for both the positive (10 ng/mL IL-8) and negative (0 ng/mL IL-8) samples (n = 3). **(B)** We also tested 15 min and 1 h for PEA probe binding. 15-minute immunobinding generated comparable ΔCt value with 1-h, despite of an overall Ct value delay for both the positive and negative samples (n = 3). **(C)** MagPEA using POEGMA beads showed comparable ΔCt value in comparison to our previously developed MagPEA using carboxyl beads (n = 3). **(D)** Dose-response curves for IL-8 protein spiked into FBS, measured using three different batches of POEGMA beads. The black lines represent the four-parameter logistic (4PL) fit. The signals from NTCs are shown on the left for each curve. Error bars indicate the standard deviations from two replicate tests. **(E)** LOD of POEGMA beads-based MagPEA is orders of magnitude lower than conventional ELISA, comparable with our previous MagPEA, and close to dELISA. * represents the averaged LOD obtained from three replicates.

We optimized the POEGMA bead-based MagPEA by using IL-8 as a model target. Specifically, IL-8 was selected as a representative target protein due to its clinical relevance in various inflammatory conditions (Russo et al., 2014). For example, IL-8 has previously been identified as a biomarker for predicting the severity of COVID-19 (Del Valle et al., 2020). Besides the elimination of beads pre-blocking and extensive washing steps, we explored whether the overall efficiency of our MagPEA workflow could be enhanced by shortening the immunobinding times. For this purpose, recombinant IL-8 protein spike-ins at final concentrations of 10 ng/mL and 0 ng/mL in 20% FBS served as the positive and negative controls, respectively, and their ΔC_t_ values were calculated to determine the optimal assay conditions. For the first immunobinding step, our results showed that decreasing the time from 1 h to 15 min did not significantly affect the signal for both the positive and negative controls (Figure 5A, Student’s t-test on ΔC_t_, *p* = 0.84). For the second immunobinding step, decreasing the time from 1 h to 15 min resulted in an overall Ct value delay for both the positive and negative controls, but the ΔCt value was not significantly affected (Figure 5B, Student’s t-test on ΔC_t_, *p* = 0.97). With these optimized conditions, the MagPEA workflow using POEGMA beads can be finished within 40 min. In addition, we evaluated the impact of POEGMA beads on PCR amplification efficiency by spiking varying amounts of beads, ranging from 10^5^ to 5 × 10^6^ beads, into PCR reaction mix containing DNA templates. We found PCR inhibition only with 5 × 10^6^ beads, suggesting a good compatibility between POEGMA beads with PCR reaction at a reasonable bead input (Supplementary Figure S2).

We next characterized the assay performance for IL-8 detection using the optimized assay conditions. Using 10 ng/mL and 0 ng/mL of IL-8 protein spiked in 20% FBS, the POEGMA beads-based MagPEA achieved comparable ΔC_t_ values compared to our previously developed carboxyl beads-based MagPEA (Student’s t-test on ΔC_t_, *p* = 0.80), showing the effectiveness of POEGMA beads in streamlining the MagPEA workflow while maintaining good assay sensitivity (Figure 5C). Moreover, using three different batches of POEGMA beads, we tested serially diluted IL-8 to establish standard curves and determine the limit-of-detection (LOD) using our assay (Figure 5D). Specifically, LOD was obtained by using the mean C_t_ value of no-target control (NTC) minus 3-fold standard deviation (SD) as threshold, followed by back-calculation via the standard curve. We achieved LODs of 27, 11.7, and 16.8 fg/mL across three tests, confirming the high reproducibility of our approach. We also evaluated the signal variability in our assay by calculating the intra-assay coefficient of variation (CV) across three independent tests at each target concentration. As shown in Supplementary Figure S3, our assay demonstrated an intra-assay CV range of 0.29%–3.23%, highlighting the assay’s high precision and robustness in the calculated LOD. The averaged LOD is 18.5 fg/mL, which is ∼500-fold lower than that of the widely used Quantikine ELISA kit (R&D Systems) and comparable to the LOD of our previously developed MagPEA (Zhang et al., 2022b) and dELISA (Wu et al., 2022), both targeting IL-8 and utilizing similar LOD calculation approaches, but our approach involves a significantly simplified and shortened assay workflow (Figure 5E). Similarly, we also determined the limit of quantification (LOQ) of our assay by setting a threshold of 10-fold SD above the background signal, resulting in an averaged LOQ of 54.7 fg/mL. Moreover, our assay showed a wide dynamic range across 7 orders of magnitude. Overall, incorporating POEGMA beads into MagPEA workflow significantly enhanced assay simplicity, providing an effective and robust solution for highly sensitive protein detection.

## Discussion

In this work, we developed a streamlined MagPEA using magnetic beads coated with antifouling POEGMA polymer brushes for highly sensitive protein detection. Using a facile GOx-assisted ARGET-ATRP technique, we successfully synthesized POEGMA polymer brushes on bead surface under ambient environment. Using BSA as a model protein, the POEGMA beads have shown good antifouling properties in minimizing the non-specific adsorption of biomolecules, laying the foundation for obviating bead pre-blocking and washings in our assay workflow. Moreover, the porous structure of polymer brushes facilitated the efficient immobilization of capture antibody onto bead surfaces through vacuum suction, offering a promising alternative to traditional covalent coupling chemistries for antibody immobilization onto magnetic beads that heavily relies on the availability of active chemical groups. We further improved the overall efficiency of our MagPEA workflow by reducing the total immunobinding time from 2 h to 30 min. Using IL-8 as a validation, we conducted POEGMA beads-based MagPEA and showcased an assay sensitivity on par with our previously developed method and dELISA but with a significantly simplified workflow. This simplification not only reduces procedural errors during bead-based immunoassays, but also provides a practical solution for performing sensitive protein detection at a higher throughput.

The selection of BSA as the model protein during our technology development is based on a few factors. First, BSA is well-characterized and readily available in various modified forms, such as with fluorescent labels, which are crucial for initial testing. Second, BSA is both abundant and cost-effective, making it a practical choice for preliminary optimization of various experimental conditions (Kunde and Wairkar, 2022). Additionally, its properties are comparable to those of many other proteins, allowing us to gain valuable insights into how different proteins might interact with the POEGMA polymer-coated magnetic beads (Ruan et al., 2022). Specifically, BSA is commonly used in literatures as a model protein for evaluating various surface properties, such as antifouling performance and protein immobilization efficiency, on solid substrates including magnetic beads (Ma et al., 2006; van Andel et al., 2018; Zhang et al., 2022b).

Our method stands out as one of the most sensitive protein detection methods with minimal operation stringencies required during the assay workflow. Although incorporating antifouling polymer brushes onto solid substrates is an established approach to minimize non-specific signals and reduce complexity in immunoassays, previous efforts fall short in achieving high sensitivity (Hucknall et al., 2009; Joh et al., 2017; Fontes et al., 2018; Heggestad et al., 2021). This limitation may stem from the lower binding capacity of traditional solid substrates, such as glass plates or silicon wafers, despite of reduced background signals. In contrast, our assay leveraged antifouling magnetic beads as the solid substrate, offering significantly higher binding capacity and faster binding kinetics, which enhance the protein detection sensitivity while maintaining a straightforward assay procedure.

The utilization of magnetic beads in immunoassays has been extensively explored, leading to the development of various methods that can be classified into two main categories based on different signal generation schemes mediated by the molecules conjugated onto detection antibody. The first category employs enzymes to catalyze fluorogenic substrate for signal generation. Notable examples include the commercial Luminex assay and dELISA. Although the Luminex assay has exceptional multiplexing capabilities using color-coded beads, its sensitivity generally reaches the pg/mL range, comparable to traditional ELISA (Dunbar, 2006). Comparatively, dELISA offers ultrahigh sensitivity by enabling single-molecule detection, yet it necessitates sophisticated instrumentation to implement the assay, limiting its accessibility (Wilson et al., 2016; Duffy, 2023). The second category involves the use of DNA oligonucleotides for signal generation through PCR or related DNA amplification techniques. For example, immuno-PCR uses a PCR template on the detection antibody to achieve higher sensitivity than conventional ELISA due to the exponential amplification of PCR (Niemeyer et al., 2007; Chang et al., 2016). However, immuno-PCR is still subjected to a relatively high background, as non-specifically bound DNA-antibody conjugates onto beads can generate signals indistinguishable from true positive signals (Zhang et al., 2022a). To address this challenge, magnetic beads-based PLA and PEA have been developed, achieving sensitivity down to fg/mL (Darmanis et al., 2010; Zhang et al., 2022b). These methods leverage the principle of “proximity”, wherein the binding of two antibodies to the same target generates a true positive signal, thereby significantly reducing background. Nevertheless, all these beads-based methods involve cumbersome workflows, including bead pre-blocking and extensive washing steps, and typically require several hours to get the results. In contrast, our POEGMA beads-based MagPEA offers significant advantages in terms of sensitivity, simplicity, and speed. It provides a more streamlined workflow and faster results compared to existing methods, making it a promising alternative for efficient and sensitive protein detection.

There are several limitations in the current work that need further refinement in future studies. First, while this study primarily focuses on method development and characterization using BSA as a model protein and IL-8 as a proof-of-concept biomarker, expanding our approach to detect a broader range of biomarkers is essential to demonstrate its versatility. Second, we only tested IL-8 spiked into FBS that mimics the complex environment of human samples. Although FBS is a widely accept matrix to evaluate immunoassays’ performance before applying to more challenging matrices such as human plasma (Yelleswarapu et al., 2019; Zhang et al., 2022b), future validation in real human biofluids is necessary to address potential non-specific binding from other human proteins that could affect assay results. Third, while sensitivity is a key strength of our method, exploring its multiplexing capability is also important, which is potentially achievable through color-coded beads or various PCR multiplexing strategies. This will not only highlight the assay’s broader applicability but also provide insight into how the antifouling properties of the beads could be maintained to ensure high detection specificity. Moreover, while the POEGMA beads immobilized with proteins were shown to be stable for at least 3 days under 4°C, as evidenced by consistent fluorescence intensity on beads from FITC-BSA (Supplementary Figure S4), the long-term stability of beads under various storage conditions should be evaluated in the future. Finally, our current work lacks a more comprehensive characterization of the POEGMA polymer brushes on the bead surface, due to the inherent challenge to measure the micrometer-sized beads. A deeper understanding about the properties of polymer brushes, such as thickness, density, uniformity, polymer growth kinetics, and antifouling performance in more challenging matrices, will facilitate further optimizing and enhancing the robustness of our approach.

In conclusion, despite its limitations, our streamlined MagPEA assay using POEGMA beads represents a significant advancement in biomarker detection. Its simplified workflow, high sensitivity, and speed make it an appealing choice for researchers and clinicians seeking efficient and user-friendly protein analysis methods. With these advantages and future optimizations, our method holds great promise as a solution for protein detection across various applications.

## Data Availability

The raw data supporting the conclusions of this article will be made available by the authors, without undue reservation.
