# Supplementary material for: A streamlined proximity extension assay using POEGMA polymer-coated magnetic beads for enhanced protein detection
Source: Front Bioeng Biotechnol. 2024 Nov 21;12:1462203. doi: 10.3389/fbioe.2024.1462203 (PMC11617200; doi:10.3389/fbioe.2024.1462203)
Supplement: Supplementary file 1 [file DataSheet1.docx]

**Supplementary information**

**A Streamlined Proximity Extension Assay Using POEGMA Polymer-Coated Magnetic Beads for Enhanced Protein Detection**

Jiumei Hu^1, †^, Pengfei Zhang^2, †, ‡^, Fangchi Shao^2^, and Tza-Huei Wang^1, 2, 3, *^

^1^ Department of Mechanical Engineering, Johns Hopkins University, Baltimore, MD 21218, USA

^2^ Department of Biomedical Engineering, Johns Hopkins University, Baltimore, MD 21218, USA

^3^ Institute for NanoBioTechnology, Johns Hopkins University, Baltimore, MD 21218, USA

^‡^ Present address: Department of Chemical and Biological Engineering, Princeton University, Princeton, NJ 08540, USA

^*^ Corresponding author

^†^ These authors contribute equally

**Calculating the required proteins to saturate binding sites on bead surface**

The quantity of protein capable of forming a monolayer on the bead surface depends on factors like the protein's molecular weight and its relative affinity for the bead. This estimation can be achieved using the following equation:

$$S=(\frac{6}{\rho Sd})(C)$$

Here, S is the amount of protein required to saturate the binding sites on beads (unit: mg protein/g of beads). ρS and d represent the density and diameter of beads, which are 1.4 g/cm^3^ and 2.8 μm, respectively. C denotes the capacity of bead surface for a specific protein (unit: mg protein/m^2^of bead surface). For BSA and IgG, the values of C are approximately 3 mg/m^2^ and 2.5 mg/m^2^, respectively (Cantarero et al., 1980). Based on this formula, 4.59 mg BSA or 3.83 mg IgG antibody is required to saturate 1 g POEGMA beads.

In our experiments, for 5 μL of POEGMA beads at a concentration of 10^9 beads/mL (equivalent to 75 μg of beads), 0.34 μg of BSA or 0.3 μg of IgG antibody is needed to achieve sufficient protein coating on the bead surface. When performing MagPEA targeting IL-8 protein, we used a slightly higher amount of antibody (1 μg of antibody per 75 μg of POEGMA beads) to ensure extensive antibody coverage on the bead surface.

**Supplementary figures**


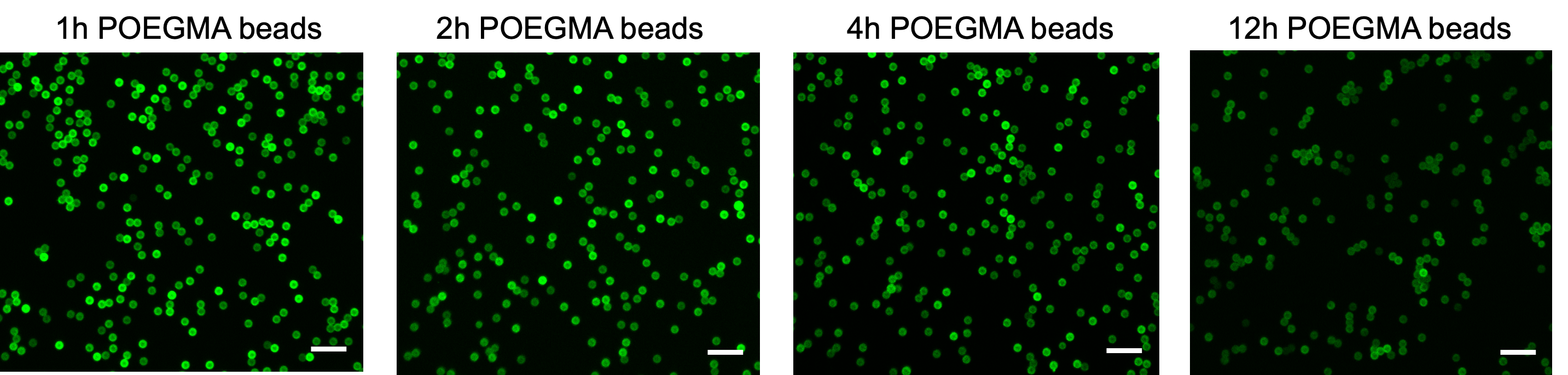


**Figure S1.** During the vacuum suction-based coating of FITC-labeled BSA onto the bead surface, microscopic images of POEGMA beads with increasing ATRP reaction time revealed a reduction in fluorescence intensity, indicating compromised protein coating efficiency with thicker POEGMA brushes. Scale bar: 10 μm.


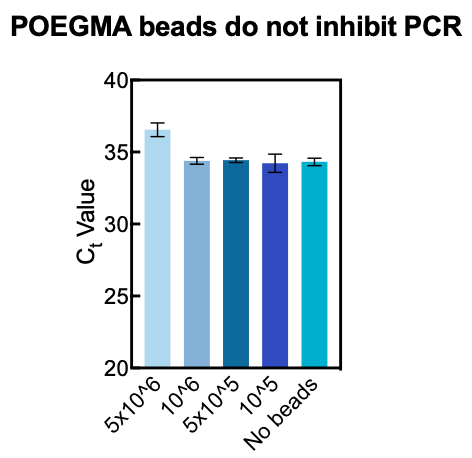


**Figure S2.** To test if POEGMA beads inhibit PCR, we spiked varying amounts of the beads in a PCR amplification buffer containing synthetic DNA. Among them, only the beads input at a quantity of 5 million showed PCR inhibition.

**
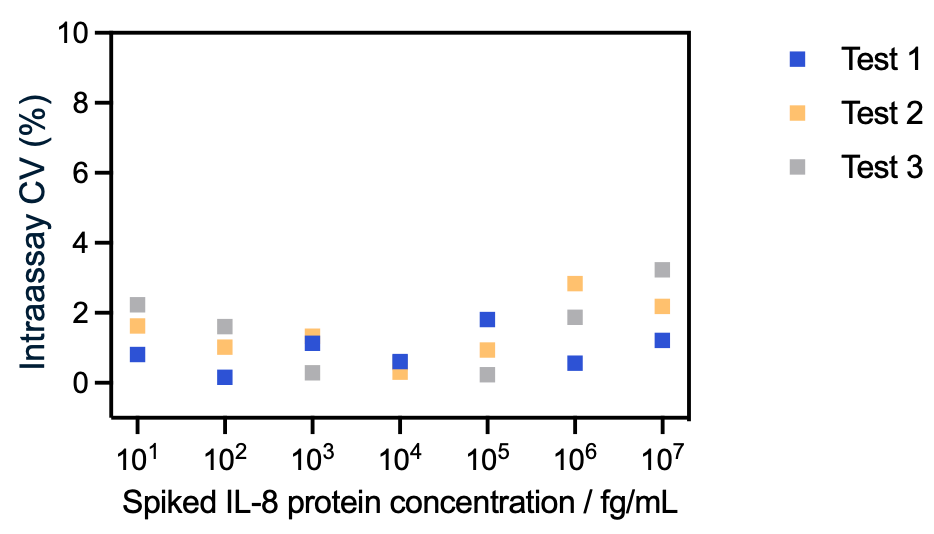
**

**Figure S3.** The intraassay CV under each condition (n=2) is below 5%, showing the high measurement consistency of our assay.

**
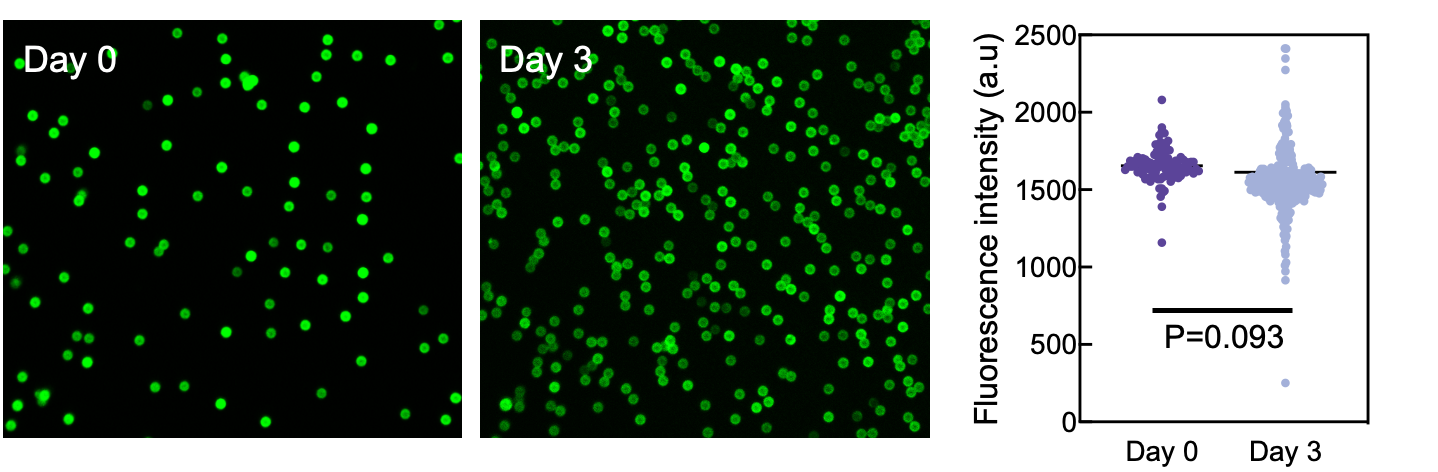
**

**Figure S4.** POEGMA beads immobilized with FITC-BSA exhibited no significant change in fluorescence intensity after 3 days of storage at 4°C (Student’s t-test, P=0.093).

**Reference**

Cantarero, L., Butler, J., and Osborne, J. (1980). The adsorptive characteristics of proteins for polystyrene and their significance in solid-phase immunoassays. *Analytical Biochemistry* 105(1)**,** 375-382.
